# Molecular detection of vector-borne pathogens from mosquitoes collected in two zoological gardens in Germany

**DOI:** 10.1007/s00436-019-06327-5

**Published:** 2019-06-01

**Authors:** Eva C. Heym, Helge Kampen, Oliver Krone, Mandy Schäfer, Doreen Werner

**Affiliations:** 1 Leibniz Centre for Agricultural Landscape Research, Eberswalder Str. 84, 15374 Muencheberg, Germany; 2 Friedrich-Loeffler-Institut, Federal Research Institute for Animal Health, Greifswald – Insel Riems, Germany; 3 Leibniz Institute for Zoo and Wildlife Research, Berlin, Germany

**Keywords:** Avian malaria, *Dirofilaria*, *Haemoproteu*s, *Leucocytozoon*, *Plasmodium*, Sindbis virus

## Abstract

In Germany, knowledge of disease agents transmitted by arthropods in zoological gardens is scarce. In the framework of ecological studies, mosquitoes were therefore collected in German zoological gardens and examined for mosquito-borne pathogen DNA and RNA. In total, 3840 mosquitoes were screened for filarial nematodes and three groups of viruses (orthobunyaviruses, flaviviruses, alphaviruses) while 405 mosquitoes were tested for avian malaria parasites. In addition to the filarial nematode species *Dirofilaria repens* (*n* = 1) and *Setaria tundra* (*n* = 8), Sindbis virus (*n* = 1) and the haemosporidian genera *Haemoproteus* (*n* = 8), *Leucocytozoon* (*n* = 10) and *Plasmodium* (*n* = 1) were demonstrated. Identified pathogens have the potential to cause disease in zoo and wild animals, but some of them also in humans. Positive mosquitoes were collected most often in July, indicating the highest infection risk during this month. Most of the pathogens were found in mosquito specimens of the *Culex pipiens* complex, suggesting that its members possibly act as the most important vectors in the surveyed zoos, although the mere demonstration of pathogen DNA/RNA in a homogenised complete mosquito is not finally indicative for a vector role. Outcomes of the study are not only significant for arthropod management in zoological gardens, but also for the general understanding of the occurrence and spread of mosquito-borne disease agents.

## Introduction

Through globalisation, both invasive mosquito species and mosquito-borne pathogens can be introduced into temperate climate zones such as central Europe. Climate change might facilitate survival of the mosquitoes in the newly invaded regions but also lead to conditions allowing pathogen development or increasing the efficiency of pathogen replication in vector-competent mosquito species (Weissenböck et al. 2010). As both globalisation and climate change significantly increase the risk of mosquito-borne disease outbreaks (Suk 2017), surveillance of mosquitoes and mosquito-borne pathogens become more and more important.

Monitoring mosquito-borne pathogens faces difficulties since not all potential vectors are known, and most infections, particularly of animals, remain undetected. This is different in zoological gardens, where mosquito-borne diseases are likely to be detected in an early stage as the health status of zoo animals is regularly and thoroughly monitored. In addition, stressed captive vertebrate species can be vulnerable for disease agents, rendering the zoo animals into sentinels for mosquito-borne diseases in urban settings (McNamara 2007). In parallel, zoological gardens offer ideal living conditions for mosquitoes through numerous breeding and resting sites and provision of a high diversity of vertebrate species for blood feeding (Tuten 2011).

Mosquito-borne diseases have been repeatedly documented from zoo animals worldwide (Adler et al. 2011). The agents of these include viruses, filarial nematodes and avian malaria parasites infecting non-adapted animals. For example, the first avian infection by *Dirofilaria immitis* Leidy, 1856 was from a Humboldt penguin living in a zoo in Japan (Sano et al. 2005). Likewise, the avian malaria parasites *Plasmodium relictum* Grassi & Feletti, 1891 and *Plasmodium elongatum* Huff, 1930 were involved in various fatal cases in zoos (e.g. Graczyk et al. 1994; Bueno et al. 2010; Sijbranda et al. 2017).

In addition to known pathogens infecting vulnerable zoo animals, pathogens previously not known to occur in the studied area have been documented from diseased animals in zoos. In 1999, for example, the Bronx Zoo/Wildlife Conservation Park, New York, was one of the first institutions recognising unusually high bird mortality that was later attributed to West Nile virus (WNV), which was not known from the USA at that time (Ludwig et al. 2002). Also in Germany, the first documentation ever of WNV was from zoological gardens, where six fatal cases of infection occurred in wild and captive birds (Ziegler et al. 2018). In the Zoological Garden Berlin, Germany, a Usutu virus strain was detected in two deceased juvenile great grey owls, which could be distinguished from strains previously known to circulate in Germany, demonstrating the mobility of mosquito-borne viruses (Ziegler et al. 2016).

Other mosquito-borne viruses documented for Germany are Batai virus (BATV), Sindbis virus (SINV) and Ťahyňa virus (TAHV) (Pilaski 1987; Jöst et al. 2010, 2011). BATV occurs widespread in Europe and was the most often detected virus in mosquitoes collected in Germany from 2011 to 2016 (Scheuch et al. 2018). Human infections by BATV seem to be rare and associated with mild symptoms, but little is known on the pathogenicity to animals (Hubálek 2008). In contrast, SINV can cause acute disease in humans and birds (Hubálek 2008; Adouchief et al. 2016). Passeriform birds are the main vertebrate hosts, but the virus has occasionally been isolated also from rodents and amphibians (Hubálek 2008). According to antibody prevalence studies in humans, TAHV is among the most common California group viruses in Eurasia (Gratz 2006), although infection rates in hare, which are the principal vertebrate hosts next to hedgehogs and rodents, are low in Germany (Dobler et al. 2006). Whether infections produce disease in animals is unknown but humans may develop influenza-like symptoms (Hubálek 2008).

The first evidence of an autochthonous *Dirofilaria* infection from Germany is from 2004, when *Dirofilaria repens* Railliet & Henry, 1911 was isolated from a diseased dog which had never left Germany (Hermosilla et al. 2006). *Dirofilaria repens* was later also demonstrated in mosquitoes from Germany (Czajka et al. 2014; Kronefeld et al. 2014a), just as *Dirofilaria immitis*, the latter being the first evidence of this nematode in mosquitoes collected outside the Mediterranean (Kronefeld et al. 2014a). *Dirofilaria repens* and *D. immitis* are endemic in southern Europe, where they mainly infect canines, felines and other carnivores, but human infections have increasingly been reported (Genchi et al. 2011). Due to improving climatic conditions, but also through the introduction of dogs and cats from endemic countries, both nematode species have successfully established in northern and eastern European countries (Genchi et al. 2011).

The nematode *Setaria tundra* Rajewsky, 1929 is generally believed to be asymptomatic in its natural hosts, which are mainly cervids, although severe disease outbreaks have been reported in reindeer populations in Finland (Laaksonen et al. 2007). There are no documented human infections with *S. tundra*.

Avian malaria is a parasitic disease caused by haemosporidian protozoans. Haemosporidians infecting birds include the genera *Plasmodium* Marchiafava & Celli, 1885, *Haemoproteus* Kruse, 1890 and *Leucocytozoon* Ziemann, 1898. There is little information about haemosporidian prevalence in Germany, although all three genera were previously documented (Krone et al. 2001, 2008; Wiersch et al. 2007). While infections of native birds with indigenous haemosporidian strains are usually harmless or asymptomatic, mortality rates are high in infected captive non-native birds which are immunologically naive (Huijben et al. 2007).

Although mosquito-associated pathogens have been detected repeatedly in Germany, it remains difficult to assess transmission risks at a local level. A recent study about the mosquito fauna of two zoological gardens in Germany showed that differences in mosquito species composition can occur even within small geographic scales (Heym et al.). Due to varying biological characteristics of the most frequent mosquito species, different pathogens could therefore become locally relevant. Additionally, blood meal analyses in the same zoological gardens demonstrated that humans and captive zoo animals were the most frequent blood-hosts of the collected mosquito species (Heym et al. 2019), suggesting that transmission of circulating mosquito-borne microorganisms between zoo animals and humans cannot be excluded, although most of them might be non-pathogenic to humans.

To better estimate the transmission risk of mosquito-borne pathogens in a zoo setting, the aim of this study was to analyse pathogen species/group, time and locality of circulation, as well as mosquito species carrying them.

## Material and methods

### Study locations and mosquito collection

Mosquitoes were collected in a 4-week rhythm from May to September 2016 and from April to September 2017 in the Tierpark Berlin (Berlin, Germany, N 52° 49.8406′, E 13° 53.0210′) and the Zoological Garden Eberswalde (Brandenburg, Germany, N 52° 82.2664′, E 13° 78.3025′). The Tierpark Berlin covers an area of ca. 160 ha, is surrounded by urban area and harbours some 7500 animals. The Zoological Garden Eberswalde comprises only 15 ha, is located in a forested area and is home to ca. 1500 animals.

Mosquito collections were conducted with eight EVS-traps (BioQuip Products, CA, USA; Rohe and Fall 1979), placed at comparable locations in both zoos at 1.6–2 m height, with a minimum of 50 m distance to ensure independence. EVS-traps were baited with dry ice producing CO_2_ as an attractant and were operated 24 h per field visit. In the Zoological Garden Eberswalde, EVS-trapping was not possible in September 2016.

In addition, adult mosquitoes were collected from their resting sites using a battery-powered Improved Prokopack Aspirator (model 1419; John W. Hock, FL, USA). Aspiration took place at a total of 15 resting sites per zoo, which were sampled once during every zoo visit for 5 min each. Resting sites consisted of shaded hiding places, which included understorey vegetation as well as eaves and wooden or stone constructions. Sampled sites were at a height of 1–3 m. Mosquitoes trying to bite the collector during fieldwork were also captured, later defined as ‘hand catches’. All collected mosquito specimens were conserved on dry ice and stored frozen at −80 °C.

### Mosquito identification

Mosquito species determination was conducted morphologically using the identification keys by Schaffner et al. (2001) and Becker et al. (2010).

Specimens belonging to the *Anopheles maculipennis* and *Culex pipiens* species complexes (*An. maculipennis* s.l., *Cx. pipiens* s.l.) were identified genetically. Species-specific ITS2-PCR was conducted to analyse *An. maculipennis* complex specimens (Proft et al. 1999; Kronefeld et al. 2014b). *Culex pipiens* complex specimens were determined by a multiplex real-time PCR assay (Rudolf et al. 2013).

Mosquitoes not belonging to a species complex, but not identifiable morphologically due to missing identification cues, were subjected to COI (cytochrome oxidase gene subunit 1) barcoding (Folmer et al. 1994; Hébert et al. 2003).

Females belonging to the *Aedes cinereus* Meigen, 1818 and *Aedes annulipes* Meigen, 1830 groups, where species identification is possible neither morphologically nor genetically, were evaluated at the group level. Also, *Culiseta morsitans* Theobald, 1901 and *Culiseta fumipennis* Stephens, 1825 females were not separated in the evaluation, due to not being reliably distinguishable morphologically or genetically.

### Pathogen screening

#### Virus diagnostics

Mosquito pools were screened using quantitative real-time PCRs following the protocols of Lambert and Lanciotti (2009) for orthobunyaviruses, Chao et al. (2007) for flaviviruses and Eshoo et al. (2007) for alphaviruses.

#### Filarial diagnostics

Mosquito screening for filarial nematodes was performed with a filarioid-specific real-time PCR assay according to Kronefeld et al. (2014a), targeting a 90 bp fragment of the mitochondrial 16S rRNA gene. Real-time PCR dissociation curves were analysed with the BioRad CFX-Manager software (www.bio-rad.com). If a signal was detected, a second conventional PCR was conducted on the sample, targeting 650 bp of the filarioid COI gene (Casiraghi et al. 2001). The resulting PCR products were processed and sequenced as described by Kronefeld et al. (2014a). Species identification of obtained sequences was conducted by standard BLAST programme search against the GenBank nucleic acid sequence database (Altschul et al. 1990).

#### Haemosporidian diagnostics

Screening for haemosporidian parasites of the genera *Haemoproteus*, *Leucocytozoon* and *Plasmodium* was performed with a real-time PCR assay targeting a 182 bp fragment of mitochondrial rDNA followed by high-resolution melting-analysis (Bell et al. 2015). Briefly, DNA was prepared from individual engorged mosquitoes using the QIAamp DNA Blood Mini Kit (Qiagen). Five microlitre of DNA was then used as a template in a 25 μl amplification reaction using the 2× QuantiTect SYBR Green PCR Master Mix (Applied Biosystems, Germany), as well as 0.4 μM forward (R330F) and reverse primer (R480RL), 8 μl sterile RNase-free water and 5 μl control or test DNA. Each real-time PCR assay systematically included a no template control (NTC) reaction and two positive control reactions (DNA from *Plasmodium ovale* Stephens, 1922 and *Haemoproteus* sp.) run in parallel with the test samples. Reactions started with an incubation step at 95 °C for 15 min, followed by 39 cycles at 94 °C for 15 s, 51 °C for 30 s and 72 °C for 30 s, and were completed by a dissociation curve ranging from 60 to 95 °C in steps of 5 °C. All data were analysed using the BioRad CFX-Manager software.

To confirm positive PCR results, two nested PCRs targeting the cytochrome b gene were conducted, which amplify a 477 bp fragment in the case of *Haemoproteus*/*Plasmodium* and a 526 bp fragment in the case of *Leucocytozoon* (Bell et al. 2015). PCR products were visualised by electrophoresis on a 1.5% agarose gel, excised and purified by means of the QIAquick PCR Purification Kit (Qiagen, Germany). This was followed by one-directional sequencing with the BigDye Terminator v1.1 Cycle Sequencing Kit (Applied Biosystems) using the primer FIFI for sequencing *Haemoproteus* and *Plasmodium* DNA and the primer L545F for *Leucocytozoon* DNA (Bell et al. 2015). Products were then purified by SigmaSpin Sequencing Reaction Clean-Up Columns (Sigma Aldrich, Germany) before loading onto a 3130 Genetic Analyser (Applied Biosystems). The genus assignment of the unknown samples was determined by comparing with sequences at GenBank.

## Results

From the Zoological Garden Eberswalde, 2407 mosquito females belonging to 20 taxa were screened for filarial nematodes and viruses. Of these, 265 blood-fed specimens, including 17 taxa, were additionally screened for avian malaria parasites.

From the Tierpark Berlin, a total of 1402 mosquito females belonging to 16 taxa were analysed for filarial nematodes and viruses. The haemosporidian screening was conducted on 140 blood-fed specimens belonging to 12 taxa. Table 1 gives an overview of the collected mosquito species and analysed specimens.

**Table 1:** Analysed number of specimens per mosquito species collected in the two sampled German zoos

	Tierpark Berlin	Zoological Garden Eberswalde
Species	Total no. of specimens (no. of blood-fed specimens) analysed	Total no. of specimens (no. of blood-fed specimens) analysed
*Ae. annulipes* group^1^	8 (0)	699 (88)
*Ae. caspius*	0 (0)	1 (0)
*Ae. cataphylla*	1 (0)	6 (2)
*Ae. cinereus* group^1^	7 (1)	44 (5)
*Ae. punctor*	0 (0)	15 (0)
*Ae. rusticus*	0 (0)	1 (0)
*Ae. sticticus*	10 (0)	10 (3)
*Ae. vexans*	352 (6)	29 (8)
*An. claviger*	2 (0)	117 (32)
*An. maculipennis* complex^2^	175 (24)	4 (3)
*An. daciae*	2 (0)	46 (15)
*An. maculipennis* s.s.	245 (47)	89 (27)
*An. messeae*	7 (2)	98 (15)
*An. plumbeus*	15 (4)	4 (1)
*Cq. richiardii*	1 (1)	98 (0)
*Cs. annulata*	142 (21)	290 (37)
*Cs. morsitans/fumipennis* ^1^	0 (0)	42 (2)
*Cx. pipiens* complex^2^	75 (8)	504 (7)
*Cx. p.* biotype *molestus*	2 (0)	0 (0)
*Cx. p.* biotype *pipiens*	305 (21)	202 (16)
*Cx. torrentium*	34 (2)	53 (2)
*Cx. modestus*	3 (0)	2 (0)
*Cx. territans*	16 (3)	53 (2)
Total no. of species	17	20
Total no. of specimens analysed	1402 (140)	2407 (265)

^1^Reliable morphological or genetic differentiation not possible

^2^Not identified to species

### Filarial nematodes

In the Zoological Garden Eberswalde, filarial nematode DNA was detected in four different mosquito species (Table 2). In addition to one nematode which could not be determined to species level, *D. repens* and *S. tundra* were identified*. Dirofilaria repens* was found in *Anopheles messeae* Falleroni, 1926 in July 2016, while evidence for *S. tundra* came from *Ae. annulipes* group mosquitoes, which had been caught in June and August 2016.

**Table 2:** Mosquito-borne pathogens found in mosquitoes in the two sampled German zoos (mosquito collections May–September 2016, April–September 2017)

Pathogen	Mosquito species	Number of specimens/pools tested positive	Collection month, year	Location^1,2^
Filarial nematodes				
*Dirofilaria repens*	*An. messeae*	1	July 2016	ZE
Filarioidea sp.	*Cx. torrentium*	1	June 2016	ZE
Filarioidea sp.	*Cx. pipiens* biotype *pipiens*	1	June 2016	TB
*Setaria tundra*	*Ae. annulipes* group	2 pools (*n* = 5, 7)	June 2016	ZE
		1	August 2016	ZE
* Setaria tundra*	*Ae. vexans*	5 pools (*n* = 3, 10, 10, 14, 7)	July 2016	TB
Viruses				
Sindbis virus	*Cx. pipiens* complex	1 pool (*n* = 12)	July 2016	TB
Haemosporidia				
*Haemoproteus* sp.	*Cx. pipiens* biotype *pipiens*	1	July 2017	TB
*Haemoproteus* sp.	*Cx. torrentium*	1	July 2017	TP
*Haemoproteus* sp.	*Cx. pipiens* biotype *pipiens*	1	July 2016	ZE
*Haemoproteus* sp.	*Cx. pipiens* biotype *pipiens*	2	August 2017	ZE
*Leucocytozoon* sp.	*Cx. pipiens* biotype *pipiens*	1	June 2016	TB
*Leucocytozoon* sp.	*Cx. pipiens* biotype *pipiens*	2	June 2017	TB
*Leucocytozoon* sp.	*Cx. pipiens* biotype *pipiens*	1	September 2017	TB
*Leucocytozoon* sp.	*Cx. torrentium*	1	June 2017	TB
*Leucocytozoon* sp	*An. maculipennis* complex	1	July 2016	ZE
*Haemoproteus* sp./*Leucocytozoon* sp. co-infection	*Cx. pipiens* biotype *pipiens*	1	May 2017	TB
*Haemoproteus* sp./*Leucocytozoon* sp. co-infection	*Cx. pipiens* biotype *pipiens*	1	July 2017	TB
*Haemoproteus* sp./*Leucocytozoon* sp. co-infection	*Cx. pipiens* biotype *pipiens*	1	August 2017	ZE
*Plasmodium* sp./*Leucocytozoon* sp. co-infection	*Cx. pipiens* biotype *pipiens*	1	June 2016	TB

^1^*ZE*, Zoological Garden Eberswalde

^2^*TB*, Tierpark Berlin

*Setaria tundra* was also detected in five *Aedes vexans* Meigen, 1830 pools collected in the Tierpark Berlin in July 2016. In addition, a *Cx. pipiens* biotype *pipiens* Linnaeus, 1758 specimen was found positive for a filarial nematode that could not be identified to species level (Table 2).

No filarial nematode DNA could be detected in mosquitoes collected in 2017.

### Mosquito-borne viruses

SINV-RNA was demonstrated in July 2016 in the Tierpark Berlin in a mosquito pool (12 individuals) belonging to the *Cx. pipiens* complex (Table 2). Mosquito-borne viruses were neither detected in mosquitoes collected in 2016 in the Zoological Garden Eberswalde nor in 2017 in any of the zoos.

### Haemosporidian protozoans

DNA of haemosporidian parasites of the genera *Haemoproteus*, *Leucocytozoon* and *Plasmodium* was demonstrated in mosquitoes from both zoos (Table 2).

*Haemoproteus* sp. DNA was detected in the Zoological Garden Eberswalde in one *Cx. pipiens* biotype *pipiens* mosquito collected in July 2016 and two *Cx. pipiens* biotype *pipiens* mosquitoes collected in August 2017 (Table 2). *Haemoproteus* sp. was also identified in one *Cx. pipiens* biotype *pipiens* and one *Cx. torrentium* Martini, 1925 specimen, respectively, collected in the Tierpark Berlin in 2017.

*Leucocytozoon* sp. was demonstrated in the Zoological Garden Eberswalde in a mosquito belonging to the *An. maculipennis* complex (Table 2). The positive sample originated from June 2016. In the Tierpark Berlin, *Leucocytozoon* sp. was detected in one *Cx. pipiens* biotype *pipiens* mosquito collected in 2016 and in three *Cx. pipiens* biotype *pipiens* mosquitoes and one *Culex torrentium* collected in 2017. The positive *Cx. pipiens* biotype *pipiens* mosquito collected in June 2016 in the Tierpark Berlin was co-infected with *Haemoproteus* sp. and *Plasmodium* sp. (Table 2), while three *Cx. pipiens* biotype *pipiens* mosquitoes collected in May and July 2017 were found co-infected with *Haemoproteus* sp. and *Leucocytozoon* sp. One *Cx. pipiens* biotype *pipiens* co-infected with *Haemoproteus* sp. and *Leucocytozoon* sp. from the Zoological Garden Eberswalde was collected in August 2017.

## Discussion

In the past, infections in zoo animals were mostly imported together with their hosts, which had been captured in the field (Canavan 1929). Nowadays, zoos are increasingly able to maintain their animal collections by own breeding or exchange with other wildlife parks, indicating linkage of potential infections to transmission within the zoo area. By analysing haematophagous arthropods in the zoo area, it can be examined to what extent vector-borne pathogens circulate, even if there are no acute disease cases. Knowing circulating disease agents helps better assess risks that humans and animals are exposed to in zoological gardens.

While *D. repens* has not been documented from a zoo animal yet, the parasite had already been detected in Germany in 2011 and 2012 in mosquitoes caught only 30 km away from the Zoological Garden Eberswalde (Czajka et al. 2014), and, apparently, autochthonous infections had been diagnosed in dogs in the same federal state of Brandenburg (Sassnau et al. 2009). An established transmission cycle of this filarial species in that area can therefore be assumed. It is conceivable, that the nematode found in this study came from a fox, which is a possible reservoir host (Magi et al. 2008). At this point, it is unclear if zoo animals in the Zoological Garden Eberswalde are asymptomatically infected with *D. repens*, as happened to a snow leopard in Japan infected by *D. immitis* (Murata et al. 2003). Awareness for *D. repens* is needed not only regarding the zoo animals but also regarding zoo visitors since this filarial species is the main agent of human dirofilariasis in Europe (Gratz 2004). Principal vectors of *D. repens* are supposed to be *Aedes albopictus* Skuse, 1895, *Cx. pipiens* s.l. and *An. maculipennis* s.l. (Cancrini and Gabrielli 2007), the latter including *An. messeae*, a species found infected in this study and shown to feed on humans in a previous study in the Zoological Garden Eberswalde (Heym et al. 2019).

The main vectors of *S. tundra* are *Aedes* species (Laaksonen et al. 2009), corresponding with the finding of the worm in the *Ae. annulipes* group in this study. However, *S. tundra* has been shown to be not very vector-specific, which could increase the probability of the nematode to expand its geographical range (Laaksonen et al. 2009). In Germany, the worm was detected in mosquitoes collected in the federal states of Baden-Wuerttemberg, Bavaria, Rhineland-Palatinate and Saxony (Czajka et al. 2012; Kronefeld et al. 2014a). Despite no known *S. tundra* infection of a zoo animal, there is a certain disease risk for cervid zoo animals if infected with the nematode at higher doses, which had led to an outbreak of peritonitis in reindeer in Finland (Laaksonen et al. 2007).

All filarial species that could not be identified in the present study were demonstrated in *Culex* species collected in June 2016. This is in agreement with the study of Czajka et al. (2012), who had also found indeterminable filarial species exclusively in *Culex* mosquitoes. Due to the ornithophilic blood feeding behaviour of most *Culex* species, Czajka et al. (2012) assume that these species most likely use birds as vertebrate hosts. This might also apply to the findings of this study, indicating that there are probably numerous unrecognised filarial species in the field, including some with disease potential for exotic birds held captive in zoos.

SINV had previously been isolated in Germany from *Cx. pipiens* s.l. and *An. maculipennis* s.l. mosquitoes collected in 2009, 2013 and 2015 (Jöst et al. 2010; Scheuch et al. 2018). The detection of SINV in a mosquito pool in the Tierpark Berlin was from a July sample, which is consistent with Jöst et al. (2010), who measured the highest mosquito infection rate with SINV in Germany in the same month in 2009. Main hosts of SINV are migratory birds, which could spread the virus over long distances (Jöst et al. 2010). For this reason, it is likely that the mosquitoes tested positive in this study had obtained the virus from a wild bird and not from the zoo animal population. Although there are no SINV cases of captive animals documented until now, the potential of the virus to infect humans makes this pathogen an important arbovirus in Germany.

Since most studies on avian malaria parasites focus on vertebrate hosts, evidence of these blood parasites from invertebrate species is of major importance to better understand disease epidemiology. Avian malaria parasites of the genus *Haemoproteus* are the most common and least pathogenic haemosporidians infecting wild birds (Atkinson and van Riper 1991). Transmission of *Haemoproteus* species has been shown to be continuous throughout the mosquito season, although main vectors are supposed to be biting midges (Atkinson and van Riper 1991). The detection of *Haemoproteus* sp. in this study is the first documentation of this protozoan genus from German mosquitoes. A study by Valkiunas et al. (2013), analysing *Aedes cantans* Meigen, 1818, has shown that *Haemoproteus* species indeed undergo sexual processes in mosquitoes, and DNA can be detected in the head, thorax and abdomen of an infected mosquito, but sporogonic development is terminated in the oocyst stage without the formation of sporozoites. Gutiérrez-López et al. (2016) demonstrated *Cx. pipiens* to be a competent vector of avian plasmodia, but not of *Haemoproteus* sp.

The blood parasite *Leucocytozoon* is known to infect mainly domestic poultry and waterfowl (Atkinson and van Riper 1991), but was recently also detected at a very high prevalence rate (85.3%) in crows from southern Germany (Schmid et al. 2017). Infections occur mainly in spring and fall (Atkinson and van Riper 1991), corresponding to the demonstration of *Leucocytozoon* sp. in this study in June and September. Similar to *Haemoproteus* sp., mosquitoes are no proven vectors of *Leucocytozoon* sp.; instead, black flies are considered the main vectors (Atkinson and van Riper 1991).

*Haemoproteus* sp. and *Leucocytozoon* sp. were the only pathogens in this study documented in both project years, indicating a high prevalence of the parasites at both locations. Both groups of parasites had been detected in birds from zoological gardens before, and a study conducted in the Oklahoma City zoo revealed that 14% of wild and captive bird species harboured them (Halpern and Bennett 1983; Chagas et al. 2016). Also in the Zoological Garden Eberswalde, massive infections of snow owls with species of the *Haemoproteus* subgenus *Parahaemoproteus* had previously occurred, subsequently resulting in regular malaria prophylaxis in owls and penguins (Valentin et al. 1994).

Avian malaria parasites of the genus *Plasmodium* were only detected in the Tierpark Berlin in 2016. Main vectors of avian *Plasmodium* species are *Culex* mosquitoes (Huijben et al. 2007), in which the parasite was also detected in this study. *Plasmodium* infections in evolutionarily adapted wild birds appear to be relatively harmless, but infections of captive non-adapted birds are often fatal (Huijben et al. 2007). Thus, avian malaria is one of the major causes of captive penguin mortality (Grilo et al. 2016). Infections of penguins are a worldwide problem in zoos, with recent reports from Japan (Ejiri et al. 2009), Brazil (Bueno et al. 2010) and Israel (Lublin et al. 2018). Also in the Tierpark Berlin, avian malaria is well known, and penguins are subjected to routine malaria prophylaxis.

Despite a study on blood meal patterns of mosquitoes collected in the Tierpark Berlin and the Zoological Garden Eberswalde showing low numbers of avian blood meals (Heym et al. 2019), the detection of avian pathogens in this study suggests *Culex*/avian interactions in both zoos. This is of major importance, since birds are especially important as reservoirs and amplifiers of zoonotic pathogens transmitted by mosquitoes. Hamer et al. (2012) showed that birds trapped in urban sites were more often seropositive for WNV than birds from less urban locations. This could have also been the case in the present study, as the only mosquito-borne virus was detected in the urban Tierpark Berlin. Additionally, with six mosquito pools and 11 single mosquito specimens positive for vector-borne pathogens, disease agents were more prevalent in Berlin than in Eberswalde, where two mosquito pools and eight single specimens tested positive for one or more pathogens. Among the pathogens, *D. repens* was only detected in the Zoological Garden Eberswalde, while SINV was only detected in the Tierpark Berlin, indicating that different pathogens can be important at different locations, depending on various ecosystemic factors, as already discussed by Heym et al. 

Most of the pathogen-positive mosquitoes were collected in July. This is in agreement with earlier studies that analysed mosquito-borne viruses and filarial nematodes in Germany and obtained most pathogen-positive mosquitoes from July to September (Kronefeld et al. 2014b; Scheuch et al. 2018).

Since some of the detected disease agents were only demonstrated in one of the project years, it cannot be clarified whether the pathogens are firmly established at the study sites. As no infection of a zoo animal with one of the pathogens detected in the mosquitoes was registered over the course of the project, the prevalence of circulating pathogens still seems to be rather low. As for viruses and filarial worms, this assumption is supported by other studies (Kronefeld et al. 2014a; Scheuch et al. 2018). More prevalent are certainly avian malaria parasites of the genera *Haemoproteus* sp. and *Leucocytozoon* sp. which were not only detected in this study in both zoos in both project years but have been repeatedly demonstrated in Germany previously from infected birds (Krone et al. 2001, 2008; Wiersch et al. 2007).

Although a relatively low number of mosquitoes were tested compared with other studies analysing mosquito-borne pathogens, it can be stated that several pathogen-positive mosquito specimens collected in the zoos were demonstrated in this study. This is of major importance, since operators of zoological gardens are pretty much aware of the risk of arthropod-borne diseases in zoo animals, such as avian malaria, but there is still little attention given to the vectors which transmit the disease-causing pathogens. In a closer collaboration between entomologists and zoological gardens, pathogen circulation could be detected in an early stage and vector control measures be implemented if considered necessary. This could be particularly relevant in Germany where mosquito-borne pathogens and their vectors have been neglected for decades. Only recently, activities to screen potential vertebrate hosts and mosquitoes for disease agents have been resumed (e.g. Jöst et al. 2010, 2011a, 2011b; Michel et al. 2018; Scheuch et al. 2018). In 2018, WNV emerged in Germany for the first time, with the first evidence coming from zoos and wildlife parks (Ziegler et al. 2018). Thus, zoological gardens can be excellent locations to detect and analyse such incidents, since here potential vertebrate hosts and various mosquito species live close together within a defined space, and previous disease cases should be known. Mosquito and mosquito-borne disease agent surveillance in zoological gardens would not only help zoos to protect their animals, but could, as a by-product, also contribute to public health surveillance.

## References

[CR1] Adler PH, Tuten HC, Nelder MP (2011). Arthropods of medicoveterinary importance in zoos. Annu Rev Entomol.

[CR2] Adouchief S, Smura T, Sane J, Vapalahti O, Kurkela S (2016). Sindbis virus as a human pathogen – epidemiology, clinical picture and pathogenesis. Rev Med Virol.

[CR3] Altschul SF, Gish W, Miller W, Myers EW, Lipman DJ (1990). Basic local alignment search tool. J Mol Biol.

[CR4] Atkinson CT, Van Riper C (1991) Pathogenicity and epizootiology of avian haematozoa: *Plasmodium, Leucocytozoon* and *Haemoproteu*s. In: Loye JE, Zuk M (eds) Bird-parasite interactions: ecology, evolution and behaviour. Oxford Ornithology Series, pp 19–48

[CR5] Becker N, Petrić D, Zgomba M, Boase C, Madon M, Dahl C, Kaiser A (2010). Mosquitoes and their control.

[CR6] Bell JA, Weckstein JD, Fecchio A, Tkach VV (2015). A new real-time PCR protocol for detection of avian haemosporidians. Parasit Vectors.

[CR7] Bueno MG, Lopez RPG, Menezes RMTD, Costa-Nascimento MDJ, Lima GFMDC, Araújo RDS, Guida FJV, Kirchgatter K (2010). Identification of *Plasmodium relictum* causing mortality in penguins (*Spheniscus magellanicus*) from São Paulo Zoo, Brazil. Vet Parasitol.

[CR8] Canavan WPN (1929). Nematode parasites of vertebrates in the Philadelphia Zoological Garden and vicinity. Parasitology.

[CR9] Cancrini G, Gabrielli S (2007) Vectors of *Dirofilaria* nematodes: biology, behaviour and host/parasite relationships. In: Cringoli G (ed.) *Dirofilaria immitis* and *D. repens* in dog and cat, and human infections. Mappe Parassitologiche, Naples, pp 48–59

[CR10] Casiraghi M, Anderson TJC, Bandi C, Bazzocchi C, Genchi C (2001). A phylogenetic analysis of filarial nematodes: comparison with the phylogeny of *Wolbachia* endosymbionts. Parasitology.

[CR11] Chagas CRF, Guimarães LDO, Monteiro EF, Valkiūnas G, Katayama MV, Santos SV, Guida FJV, Simões RF, Kirchgatter K (2016). Hemosporidian parasites of free-living birds in the São Paulo Zoo, Brazil. Parasitol Res.

[CR12] Chao D-Y, Davis BS, Chang GJJ (2007). Development of multiplex real-time reverse transcriptase PCR assays for detecting eight medically important flaviviruses in mosquitoes. J Clin Microbiol.

[CR13] Czajka C, Becker N, Poppert S, Jöst H, Schmidt-Chanasit J, Krüger A (2012). Molecular detection of *Setaria tundra* (Nematoda: Filarioidea) and an unidentified filarial species in mosquitoes in Germany. Parasit Vectors.

[CR14] Czajka C, Becker N, Jöst H, Poppert S, Schmidt-Chanasit J, Krüger A, Tannich E (2014). Stable transmission of *Dirofilaria repens* nematodes, northern Germany. Emerg Infect Dis.

[CR15] Dobler G, Wölfel R, Schmüser H, Essbauer S, Pfeffer M (2006). Seroprevalence of tick-borne and mosquito-borne arboviruses in European brown hares in northern and western Germany. Int J Med Microbiol.

[CR16] Ejiri H, Sato Y, Sawai R, Sasaki E, Matsumoto R, Ueda M, Higa Y, Tsuda Y, Omori S, Murata K, Yukawa M (2009). Prevalence of avian malaria parasite in mosquitoes collected at a zoological garden in Japan. Parasitol Res.

[CR17] Eshoo MW, Whitehouse CA, Zoll ST, Massire C, Pennella T-TD, Blyn LB, Sampath R, Hall TA, Ecker JA, Desai A, Wasieloski LP, Li F, Turell MJ, Schink A, Rudnick K, Otero G, Weaver SC, Ludwig GV, Hofstadler SA, Ecker DJ (2007). Direct broad-range detection of alphaviruses in mosquito extracts. Virol J.

[CR18] Folmer O, Black M, Hoeh W, Lutz R, Vrijenhoek R (1994). DNA primers for amplification of mitochondrial cytochrome c oxidase subunit I from diverse metazoan invertebrates. Mol Mar Biol Biotechnol.

[CR19] Genchi C, Kramer LH, Rivasi F (2011). Dirofilarial infections in Europe. Vector-borne Zoonot Dis.

[CR20] Graczyk TK, Cranfield MR, Mccutchan TF, Bicknese EJ (1994). Characteristics of naturally acquired avian malaria infections in naive juvenile African black-footed penguins (*Spheniscus demersus*). Parasitol Res.

[CR21] Gratz N (2004) The vector-borne human infections of Europe – their distribution and burden on public health. World Health Organization, Regional Office for Europe, Copenhagen, Denmark

[CR22] Gratz N (2006). Vector- and rodent-borne diseases in Europe and North America.

[CR23] Grilo ML, Vanstreels RE, Wallace R, Garcia-Parraga D, Braga EM, Chitty J, Catao-Dias JL, Madeira De Carvalho LM (2016). Malaria in penguins – current perceptions. Avian Pathol.

[CR24] Gutiérrez-López R, Martínez-De La Puente J, Gangoso L, Yan J, Soriguer RC, Figuerola J (2016). Do mosquitoes transmit the avian malaria-like parasite *Haemoproteus*? An experimental test of vector competence using mosquito saliva. Parasit Vectors.

[CR25] Halpern N, Bennett GF (1983). *Haemoproteus* and *Leucocytozoon* infections in birds of the Oklahoma city zoo. J Wildl Dis.

[CR26] Hamer SA, Lehrer E, Magle SB (2012). Wild birds as sentinels for multiple zoonotic pathogens along an urban to rural gradient in greater Chicago, Illinois. Zoonoses Public Health.

[CR27] Hébert PDN, Ratnasingham S, De Waard JR (2003). Barcoding animal life: cytochrome c oxidase subunit 1 divergences among closely related species. Proc R Soc Lond B Biol Sci.

[CR28] Hermosilla C, Pantchev N, Dyachenko V, Gutmann M, Bauer C (2006). First autochthonous case of canine ocular *Dirofilaria repens* infection in Germany. Vet Rec.

[CR29] Heym EC, Kampen H, Walther D (2018). Mosquito species composition and phenology (Diptera, Culicidae) in two German zoological gardens imply different risks of mosquito-borne pathogen transmission. J Vector Ecol.

[CR30] Heym EC, Kampen H, Schäfer M, Walther D (2019) Mosquito bloodmeal preferences in two zoological gardens in Germany. Med Vet Entomol 33, 203-212 10.1111/mve.12350 30474300

[CR31] Hubálek Z (2008). Mosquito-borne viruses in Europe. Parasitol Res.

[CR32] Huijben S, Schaftenaar W, Wijsman A, Paaijmans KP, Takken W (2007) Avian malaria in Europe: an emerging infectious disease? In: Takken W, Knols BG (eds) Emerging pests and vector-borne diseases in Europe. Wageningen Academic Publishers, Wageningen, Netherlands, pp 59–74

[CR33] Jöst H, Bialonski A, Storch V, Gunther S, Becker N, Schmidt-Chanasit J (2010). Isolation and phylogenetic analysis of Sindbis viruses from mosquitoes in Germany. J Clin Microbiol.

[CR34] Jöst H, Bialonski A, Schmetz C, Gunther S, Becker N, Schmidt-Chanasit J (2011b) Isolation and phylogenetic analysis of Batai virus, Germany. Am J Trop Med Hyg 84:241–243 10.4269/ajtmh.2011.10-0483 PMC3029175 21292892

[CR35] Krone O, Priemer J, Streich J, Sommer P, Langgemach T, Lessow O (2001). Haemosporida of birds of prey and owls from Germany. Acta Protozool.

[CR36] Krone O, Waldenström J, Valkiūnas G, Lessow O, Müller K, Iezhova TA, Fickel J, Bensch S (2008). Haemosporidian blood parasites (Haemosporida, Haemoproteidae) in European birds of prey and owls. J Parasitol.

[CR37] Kronefeld M, Kampen H, Sassnau R, Werner D (2014). Molecular detection of *Dirofilaria immitis*, *Dirofilaria repens* and *Setaria tundra* in mosquitoes from Germany. Parasit Vectors.

[CR38] Kronefeld M, Werner D, Kampen H (2014). PCR identification and distribution of *Anopheles daciae* (Diptera, Culicidae) in Germany. Parasitol Res.

[CR39] Laaksonen S, Kuusela J, Nikander S, Nylund M, Oksanen A (2007). Outbreak of parasitic peritonitis in reindeer in Finland. Vet Rec.

[CR40] Laaksonen S, Solismaa M, Kortet R, Kuusela J, Oksanen A (2009). Vectors and transmission dynamics for *Setaria tundra* (Filarioidea; Onchocercidae), a parasite of reindeer in Finland. Parasit Vectors.

[CR41] Lambert AJ, Lanciotti RS (2009). Consensus amplification and novel multiplex sequencing method for S segment species identification of 47 viruses of the *Orthobunyavirus, Phlebovirus* and *Nairovirus* genera of the family Bunyaviridae. J Clin Microbiol.

[CR42] Lublin A, Lapid R, Mechani S, Farnoushi Y, Goldbach C, Edery N (2018). Unusual prevalence of avian malaria (*Plasmodium* sp.) in the adult population of Humboldt penguins (*Spheniscus humboldti*) at an Israeli zoo between years 2012 and 2015. Isr J Vet Med.

[CR43] Ludwig GV, Calle PP, Mangiafico JA, Raphael BL, Danner DK, Hile JA, Clippinger TL, Smith JF, Cook RA, McNamara T (2002). An outbreak of West Nile virus in a New York city captive wildlife population. Am J Trop Med Hyg.

[CR44] Magi M, Calderini P, Gabrielli S, Dell’Omodarme M, Macchioni F, Prati MC, Cancrini G (2008). *Vulpes vulpes*: a possible wild reservoir for zoonotic filariae. Vector-borne Zoonot Dis.

[CR45] McNamara T (2007). The role of zoos in biosurveillance. Int Zoo Yearb.

[CR46] Michel F, Fischer D, Eiden M, Fast C, Reuschel M, Müller K, Rinder M, Urbaniak S, Brandes F, Schwehn R, Lühken R, Groschup MH, Ziegler U (2018). West Nile virus and Usutu virus monitoring of wild birds in Germany. Int J Environ Res Public Health.

[CR47] Murata K, Yanai T, Agatsuma T, Uni S (2003). *Dirofilaria immitis* infection of a snow leopard (*Uncia uncia*) in a Japanese zoo with mitochondrial DNA analysis. J Vet Med Sci.

[CR48] Pilaski J (1987). Contribution to the ecology of Tahyna virus in Central Europe. J Vector Ecol.

[CR49] Proft J, Maier WA, Kampen H (1999). Identification of six sibling species of the *Anopheles maculipennis* complex (Diptera: Culicidae) by a polymerase chain reaction assay. Parasitol Res.

[CR50] Rohe DL, Fall RP (1979) A miniature battery powered CO_2_ baited trap for mosquito borne encephalitis surveillance. Bull Soc Vector Ecol 4:24–27

[CR51] Rudolf M, Czajka C, Börstler J, Melaun C, Jöst H, von Thien H, Badusche M, Becker N, Schmidt-Chanasit J, Krüger A, Tannich E, Becker S (2013). First nationwide surveillance of *Culex pipiens* complex and *Culex torrentium* mosquitoes demonstrated the presence of *Culex pipiens* biotype *pipiens*/*molestus* hybrids in Germany. PLoS One.

[CR52] Sano Y, Aoki M, Takahashi H, Miura M, Komatsu M, Abe Y, Kakino J, Itagaki T (2005). The first record of *Dirofilaria immitis* infection in a Humboldt penguin, *Spheniscus humboldti*. J Parasitol.

[CR53] Sassnau R, Dyachenko V, Pantchev N, Stöckel F, Dittmar K, Daugschies A (2009) *Dirofilaria repens* infestation in a sled dog kennel in the federal state of Brandenburg (Germany). Diagnosis and therapy of canine cutaneous dirofilariosis. Tierärztl Prax 37:95–101 [In German]

[CR54] Schaffner F, Angel G, Geoffroy B, Hervy JP, Rhaiem A, Brunhes J (2001). The mosquitoes of Europe (CD-ROM). EID Méditerrannée.

[CR55] Scheuch DE, Schäfer M, Eiden M, Heym EC, Ziegler U, Walther D, Schmidt-Chanasit J, Keller M, Groschup MH, Kampen H (2018). Detection of Usutu, Sindbis and Batai viruses in mosquitoes (Diptera: Culicidae) collected in Germany, 2011-2016. Viruses.

[CR56] Schmid S, Fachet K, Dinkel A, Mackenstedt U, Woog F (2017). Carrion crows (*Corvus corone*) of southwest Germany: important hosts for haemosporidian parasites. Malar J.

[CR57] Sijbranda DC, Hunter S, Howe L, Lenting B, Argilla L, Gartrell BD (2017). Cases of mortality in little penguins (*Eudyptula minor*) in New Zealand associated with avian malaria. N Z Vet J.

[CR58] Suk J (2017). Preparedness for mosquito-borne diseases in Europe: the ECDC perspective. Eur J Pub Health.

[CR59] Tuten HC (2011) Zoos as experiment environments: biology of larval and adult mosquitoes (Diptera: Culicidae). Ph.D. thesis, Clemson University, USA

[CR60] Valentin A, Haberkorn A, Hensch B, Jakob W (1994). Massive Malaria-Infektionen mit *Parahaemoproteus* spec. in Schnee-Eulen (*Nyctea scandiaca*) und deren Behandlung mit Primaquin. Verhandlungsber Erkrank Zootiere.

[CR61] Valkiunas G, Kazlauskiene R, Bernotiene R, Palinauskas V, Iezhova TA (2013). Abortive long-lasting sporogony of two *Haemoproteus* species (Haemosporida, Haemoproteidae) in the mosquito *Ochlerotatus cantans*, with perspectives on haemosporidian vector research. Parasitol Res.

[CR62] Weissenböck H, Hubálek Z, Bakonyi T, Nowotny N (2010). Zoonotic mosquito-borne flaviviruses: worldwide presence of agents with proven pathogenicity and potential candidates of future emerging diseases. Vet Microbiol.

[CR63] Wiersch SC, Lubjuhn T, Maier WA, Kampen H (2007). Haemosporidian infection in passerine birds from Lower Saxony. J Ornithol.

[CR64] Ziegler U, Fast C, Eiden M, Bock S, Schulze C, Hoeper D, Ochs A, Schlieben P, Keller M, Zielke DE, Luehken R, Cadar D, Walther D, Schmidt-Chanasit J, Groschup MH (2016). Evidence for an independent third Usutu virus introduction into Germany. Vet Microbiol.

[CR65] Ziegler U, Lühken R, Keller M, Cadar D, van der Grinten E, Michel F, Albrecht K, Eiden M, Rinder M, Lachmann L, Höper D, Vina-Rodriguez A, Gaede W, Pohl A, Schmidt-Chanasit J, Groschup MH (2018). West Nile virus epizootic in Germany. Antivir Res.

